# Intra-tumor heterogeneity in breast cancer has limited impact on transcriptomic-based molecular profiling

**DOI:** 10.1186/s12885-017-3815-2

**Published:** 2017-11-29

**Authors:** Govindasamy-Muralidharan Karthik, Mattias Rantalainen, Gustav Stålhammar, John Lövrot, Ikram Ullah, Amjad Alkodsi, Ran Ma, Lena Wedlund, Johan Lindberg, Jan Frisell, Jonas Bergh, Johan Hartman

**Affiliations:** 1 Department of Oncology and Pathology, Karolinska Institutet, CCK, 17176 Stockholm, Sweden; 2 Department of Medical Epidemiology and Biostatistics, Karolinska Institutet, 17177 Stockholm, Sweden; 3 St. Erik Eye Hospital, 11282 Stockholm, Sweden; 4 Genome-Scale Biology Research Program Unit, Faculty of Medicine, University of Helsinki, Helsinki, Finland; 5 Department of Clinical Pathology and Cytology, Karolinska University Laboratory, Stockholm, Sweden; 6 Department of Molecular Medicine and Surgery, Karolinska University Hospital, 17176 Stockholm, Sweden; 7 Radiumhemmet – Karolinska Oncology, Karolinska University Hospital, 17176 Stockholm, Sweden; 8 Department of Clinical Science and Education, Karolinska Institutet, South General Hospital, 11883 Stockholm, Sweden

**Keywords:** Breast cancer, Molecular diagnostics, Molecular subtypes, Intra-tumor transcriptomic heterogeneity

## Abstract

**Background:**

Transcriptomic profiling of breast tumors provides opportunity for subtyping and molecular-based patient stratification. In diagnostic applications the specimen profiled should be representative of the expression profile of the whole tumor and ideally capture properties of the most aggressive part of the tumor. However, breast cancers commonly exhibit intra-tumor heterogeneity at molecular, genomic and in phenotypic level, which can arise during tumor evolution. Currently it is not established to what extent a random sampling approach may influence molecular breast cancer diagnostics.

**Methods:**

In this study we applied RNA-sequencing to quantify gene expression in 43 pieces (2-5 pieces per tumor) from 12 breast tumors (Cohort 1). We determined molecular subtype and transcriptomic grade for all tumor pieces and analysed to what extent pieces originating from the same tumors are concordant or discordant with each other. Additionally, we validated our finding in an independent cohort consisting of 19 pieces (2-6 pieces per tumor) from 6 breast tumors (Cohort 2) profiled using microarray technique. Exome sequencing was also performed on this cohort, to investigate the extent of intra-tumor genomic heterogeneity versus the intra-tumor molecular subtype classifications.

**Results:**

Molecular subtyping was consistent in 11 out of 12 tumors and transcriptomic grade assignments were consistent in 11 out of 12 tumors as well. Molecular subtype predictions revealed consistent subtypes in four out of six patients in this cohort 2. Interestingly, we observed extensive intra-tumor genomic heterogeneity in these tumor pieces but not in their molecular subtype classifications.

**Conclusions:**

Our results suggest that macroscopic intra-tumoral transcriptomic heterogeneity is limited and unlikely to have an impact on molecular diagnostics for most patients.

**Electronic supplementary material:**

The online version of this article (10.1186/s12885-017-3815-2) contains supplementary material, which is available to authorized users.

## Background

Breast cancer incidence in the industrialised countries has markedly increased during the last century but the mortality rate remains unchanged, and it has even decreased in some countries [1]. Modern adjuvant therapy is the main reason for these improvements in outcome and it is delivered based on the analysis of therapy predictive biomarkers and risk factors such as age, stage and histopathological grade. In the general diagnostic workup, expression of the therapy predictive biomarkers, such as estrogen receptor (ER), progesterone receptor (PR) and Human epidermal growth factor receptor 2 (HER2), are analysed by routine immunohistochemistry (IHC). Based on the statues of these therapy predictive markers, informed clinical decisions are taken. Apart from the traditional immunohistochemical analysis, genome-wide transcriptional profiling has provided opportunity to classify breast cancers in to distinct molecular subtypes, which have been proven to have significant prognostic value [2–5]. Various commercially available gene signature panels, such as Oncotype DX [6] Prosigna^®^ [7] and MammaPrint [8, 9] are currently available for prognostic purposes, which can predict therapy response and the likelihood of cancer recurrence. Recently, we have determined the histological grade of breast cancer using the RNA-sequencing data from 275 breast cancer patients [10]. In that study, by using RNA sequencing data, we managed to reclassify the transcriptomic grade (TG) [11] for grade 2 tumors, which is a clinically challenging group for making clinical decisions regarding therapy [12]. Multiple other studies demonstrated that combining molecular signatures with routine histopathological grading can improve prognostic power [12–15]. These data suggests that integration of gene expression-based analysis along with the routine immunohistochemical analysis will be the future in clinics for making informed clinical decisions.

It is widely acknowledged that breast cancers exhibit substantial intra-tumor heterogeneity [16–18]. In surgical pathology, breast tumor grading is commonly performed by Nottingham histological grade (NHG) criteria; taking both tumor differentiation, mitosis and nuclear atypia into account [19]. However, mitoses and atypia varies throughout the tumor area, leading to inter-observer variability in morphology assessment. Heterogeneity is also evident as different growth patterns that can be observed within the same primary tumor [20]. Several massive parallel sequencing studies have demonstrated that both spatial and temporal genomic heterogeneity are common features of breast cancer [21–24]. Hence, it is postulated that a biopsy from one topographic region of the tumor may have different gene expression profile compared to another region, which can potentially affect the utility of gene expression based molecular profiling in pathology laboratories. For a reliable transcription based molecular profiling, the methodology should be robust (irrespective of the tumor region profiled) in representing the whole tumor characteristics, and not be influenced by existing intra-tumor heterogeneity.

It is unknown to what extent the therapy predictive biomarkers and predicted molecular subtypes are displaying intra-tumor heterogeneity at transcriptomic level. The aim of this study was to investigate the transcriptional heterogeneity in primary breast cancers. Here, we have performed RNA-sequencing on multiple tumor regions of 12 primary breast cancers (43 tumor pieces). We determined the molecular subtypes and transcriptomic grade (TG) of all the tumor regions profiled. Furthermore, gene expression and IHC statuses of therapy predictive factors (ER, PR and HER2) were also investigated in these samples. These results were further validated in an independent cohort consisting of 19 tumor pieces from 6 breast cancers using microarray technology. Additionally, we also performed exome sequencing on these 19 tumor pieces to investigate intra-tumor genomic heterogeneity.

## Methods

### Patient material

During 2015, material from 12 breast cancer patients (Cohort 1) were prospectively collected at Karolinska University Hospital. From each one of these patients at least two spatially separated tumor pieces were collected and snap-frozen. In total, 43 pieces were collected (2-6 pieces per tumor). The additional retrospective validation data set, referred to as “Cohort 2” consisted of 15 breast tumors, out of which 6 tumors had multiple tumor pieces (19 tumor pieces in total, 2-6 pieces per tumor). The patients were identified through searches in the laboratory information system (FlexLab/Sympathy®, Tieto, Sweden) using the digitalized patient medical records between 2000 and 2011. For each of these patients, we collected formalin-fixed paraffin-embedded (FFPE) material from primary breast tumors. From the majority of primary tumors, multiple tumor areas of different topography were isolated (>5 mm distance from each other) resulting in 19 tumor pieces from 6 patient samples. These studies have been approved by the Regional Ethical Review Board in Stockholm (Regionala etikprövningsnämnden i Stockholm). All participants in the prospective study signed informed consent allowing for molecular profiling.

### RNA-sequencing and data analysis

RNA was extracted from fresh frozen tumors using AllPrep DNA/RNA/Protein mini kit (Qiagen). One μg of total RNA was used for rRNA depletion using RiboZero (Illumina) and stranded RNAseq libraries were constructed using TruSeq Stranded Total RNA Library Prep Kit (Illumina). RNAseq libraries to a median of 33 million read-pairs per library (paired-end 2 × 101 bases, Illumina HiSeq 2500). The detailed protocol has been published previously [10]. Pre-processing was performed using AutoSeq (https://github.com/ClinSeq/autoseq), using the same pre-processing procedure as described previously [10]. In brief, standard Illumina adapters were trimmed using skewer version 0.1.117 [25] with default parameters. Alignment was carried out using STAR aligner version 2.4.0e [26] and gene expression estimates were calculated with HTSeq count version 0.6.1 [27]. The RNAseq count data were normalised using the TMM method [28] in the *edgeR* package [29]. Molecular subtype, based on the PAM50 gene set [3], and transcriptomic grade were predicted from the RNA-sequencing data as described previously [10, 11]. ER, PR and HER2 status was assigned using a logistic regression model with the corresponding gene as predictor [10]. Principal Component Analysis (PCA) was applied using the PAM50 gene set [3] after mean centering of the variables. All statistical analyses were carried out in the R environment [30].

### IHC assessments and pathology characterisation

The whole tumor paraffin blocks were cut into 4 μm sections and immunohistochemically stained for ER, PR, HER2 and Ki-67. FFPE sections were conditioned in CC1 solution (Ventana Medical Systems, Tucson, AZ, USA) for 36 min (Ki67) to 64 min (PR) and incubated with mouse monoclonal antibodies for Ki67 (clone 30-9) and rabbit monoclonal primary antibodies for ER (clone SP1), PR (clone 1E2), and HER2 (clone 4B5) at 35 °C (HER2, all antibodies from Roche/Ventana Medical Systems, Tucson, AZ, USA) or 37 °C (others) for 16 min (Ki67) to 44 min (ER) according to the manufacturer’s instructions (Ventana, USA), and finally counterstained with hematoxylin. Board certified pathologist at Karolinska University Hospital determined the heterogeneity (difference in percentage of positive cells for the biomarker in different regions of the tumor) of ER, PR, HER2 and Ki-67 on whole tumor sections.

### Microarray and PAM50 molecular subtyping after subgroup-specific gene-centering

For validation purpose, we investigated 19 tumor pieces from six additional patients (Cohort 2, 2-6 pieces per tumor) and profiled them using microarray technology. RNA was extracted from two 10 μM sections per FFPE tumor block (19 tumor pieces from 6 breast cancer patients) using RNeasy FFPE Kit (Qiagen, CA, USA) according to manufacturer’s instructions. SensationPlus™ FFPE Amplification kit (Affymetrix, Santa Clara, CA, USA) was used to amplify the RNA and profiled in GeneChip® Human Transcriptome Array 2.0 (Affymetrix, Santa Clara, CA, USA). Probe intensities were extracted from CEL files and background corrected, normalized and summarized for probe set expression using Affymetrix Expression Console Software. PAM50 molecular subtyping [3] of each tumor sample was performed after subgroup-specific gene-centering [31]. The population based Stockholm cohort with primary breast cancer patients [32] (GEO:GSE1456) was used as training cohort. The subgroup of patients with breast cancer relapse within the first 5 years was used to mimic this cohort. All molecular subtype analysis was done in R/Bioconductor.

### Exome sequencing and data analysis

We isolated cancer DNA from eight 10 μM sections of FFPE tissues using a QIAamp DNA FFPE Tissue Kit (Qiagen, CA, USA). We used DNA from normal axillary lymph nodes FFPE tissues as Germline controls. In all cases, we followed the manufacturer’s recommended protocol. Genomic target capture was performed using the SureSelectXT2 Human All Exon V5 kit (Agilent Technologies, Santa Clara, CA, USA) and captured libraries were whole exome sequenced on an Illumina HiSeq 2500 Instrument (Illumina, San Diego, CA, USA) using 2 × 100 bp sequencing reads. Raw sequencing reads were quality and adapter trimmed with trim galore. The trimmed reads were aligned to the reference human genome (hg19) using bwa-mem. Aligned reads were sorted and marked for duplicates with Picard. Next, base quality recalibration and realignment around indels were performed using the Genome Analysis ToolKit (GATK). The achieved coverage in target regions was on average 80× (70% targeted regions with >30× coverage). All preprocessing and downstream analyses were performed within the Anduril framework for scientific data analysis [33]. We performed point mutation calling using MuTect (50). Then, to account for potential artifacts induced by formalin-fixed paraffin embedded (FFPE) samples, we filtered C > T/G > A mutations that are private to one sample and having variant allele frequency (VAF) less than 0.15. To rescue potential real mutations, we excluded, from these criteria, the variants that are reported in the COSMIC database (version 68) and variants with at least two reads supporting the variant allele in each strand. Second, we filtered shared variants that have VAF < 0.15 if the respective control sample has any number of reads supporting the variant allele. Absolute estimation of copy number alterations was performed with AscatNgs (52), which allows the estimation of ploidy and purity values for each sample (52). Genes were assigned the copy number of the most overlapping segment. Genes were called amplified if the assigned absolute copy number was larger than average ploidy multiplied by 1.5, and were call deleted if the assigned absolute copy number was less than the average sample ploidy multiplied by 0.5.

### Intra-tumor genomic heterogeneity analysis

We used variant allele frequency (VAF) of a set of 361 putative driver genes in breast cancer compiled by Yates et al. (16), derived from exome sequencing data to demonstrate intra-tumor genomic heterogeneity in the primary tumor. Before comparing VAFs of these genes across different primary blocks in a patient, we accounted for tumor purity by dividing the VAFs by corresponding purity of the tumor block. Genomic heterogeneity plots were plotted in R using ggplot2 package. We used PyClone (25) for analyzing the subclonal population structure. PyClone is based on a Bayesian clustering method, which uses a Markov chain Monte Carlo (MCMC) based framework to estimate cellular prevalence values using somatic substitution, copy number aberration and tumor purity data (estimated using AscatNGS). We used the authors’ recommended genotype-aware PyClone-beta-binomial model with all model parameters set to recommended values (the rest of the two models are genotype-naive infinite binomial mixture model and infinite beta-binomial mixture model). PyClone is implemented in Python programming language.

We used the following criteria for filtering out low-occurrence clusters.

1. A cluster was considered only if it had 10 or more mutations.
2. A cluster *sc* in a sample *s* was considered only if the mean cellular prevalence of *sc* was greater than or equal to 0.05, i.e., *sc* was present in at least 5% of the cells in s.

## Results

### Intra-tumor molecular subtype heterogeneity based on RNA-sequencing data

**Fig. 1:**
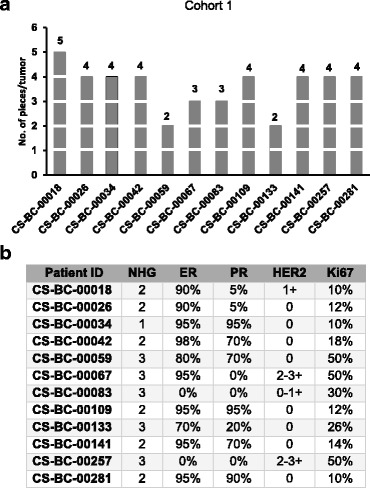
Multi-region RNA sequencing profiling cohort. **a** Bar graph illustrating the number of intra–tumor pieces analysed per breast tumor from 12 breast cancer patients. **b** The clinicopathological characteristics Nottingham grade (NHG), ER, PR, HER2 and Ki-67 status of the cohort. The values correspond to the percentage of positively stained tumor cells

**Fig. 2:**
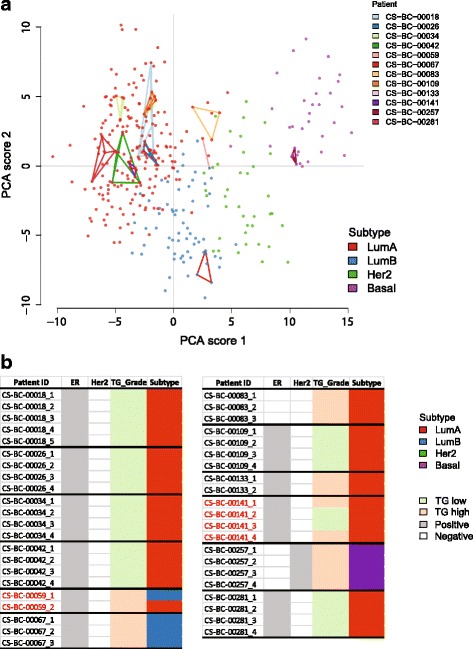
Intra-tumor molecular subtype heterogeneity (*n* = 12 tumors). **a** PCA score plot of the prospective study (points) and the heterogeneity set (points connected by lines for pieces from the same tumor) (Key: LumA = Luminal A (red colour dots), LumB = Luminal B (blue colour dots), HER2 = HER2-enriched (green colour dots), Basal = Basal-like (purple colour dots) and Normal = Normal breast-like (orange colour)). Intra-tumor pieces from each patient are connected through respective coloured lines as illustrated in the figure legend. **b** Predicted ER status, HER2 status, transcriptomic grade and molecular subtypes for all the tumor pieces (*n* = 43) are summarised in a table format. Two patient IDs are coloured in red fonts, are the ones which exhibited heterogeneous molecular subtypes or transcriptomic grades

The potential effect of intra tumor heterogeneity on molecular diagnostics was assessed in a set of 43 tumor pieces from 12 breast tumors (Cohort 1) (Fig. 1a). The routine clinicopathological data on NHG, ER, PR, HER2 and Ki-67 statuses for these 12 breast tumors are illustrated in Fig. 1b. Based on IHC, ten tumors were ER-positive/HER2-negative or positive, one tumor was HER2-positive and one tumor was triple negative (Fig. 1b). RNA-sequencing data was acquired, pre-processed and molecular subtype was predicted for each tumor piece (Fig. 2a). Consistent molecular subtypes were predicted across all pieces in 11 out of 12 tumors. In one patient (CS-BC-00059) however, one tumor piece was assigned to Luminal A, while the other tumor piece was assigned to Luminal B subtype (Fig. 2b). Based on molecular subtype analysis, our cohort consisted of 11 luminal (A/B) tumors and one basal-like tumor (Fig. 2b). We also note that in 2 patients (CS-BC-00257 and CS-BC-00083) we report discordance between IHC based subtypes and intrinsic molecular subtypes based on RNA-sequencing data. A HER2 positive tumor was classified as Basal-like subtype (CS-BC-00257) and a triple negative tumor was assigned to luminal type based on RNA sequencing data. HER2 positive individuals would generally be expected to fall into the HER2-enriched molecular subtype, however, HER2 positive samples classified as Basal-like has previously been reported [3], and we note that this particular tumor is also located in the border between basal-like and HER2-enriched subtypes in the PCA score plot (Fig. 2a). Similarly, in the PCA score plot (Fig. 2a), CS-BC-00083 is located on the border of the ‘luminal’ area (top left), and close to the HER2/Basal corner (top right). The classification model for subtype also take into account a larger gene-panel (PAM50) and not only ER, PR and HER2 statuses hence, the multivariate expression profile in this case, indicate that this tumour had the highest probability of belonging to the luminal subtype.

### Intra-tumor heterogeneity in transcriptomic grade

Transcriptomic grade (TG) is a reproducible method to divide primary breast tumors into high and low grade based on gene expression and thereby eliminate classification of tumors as intermediate-grade. TG assignments were consistent in 11 out of 12 tumors (Fig. 2b). We found minor intra-tumor differences in one patient out of 12 in respect to transcriptomic grade. In one patient (CS-BS-00141) two tumor specimens had high transcriptomic grade, and other two pieces had low grade. These results suggest that spatial heterogeneity may only have a minor impact on transcription based molecular diagnostics for most patients.

### Intra-tumor heterogeneity in biomarker expression

**Fig. 3:**
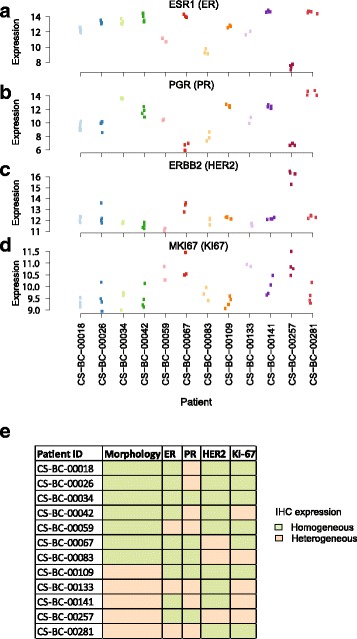
Intra tumor transcriptomic variation of *ER*, *PR*, *HER2* and *MKI67*. mRNA expression levels of (**a**) *ER* (**b**) *PR* (**c**) *HER2* and (**d**) *MKI67* across tumor pieces from 12 patients. **e** Heterogeneity assessment of whole tumor section after immunohistochemical staining of the biomarkers ER, PR HER2, Ki-67 and tumor growth patterns (morphology). Assessments were performed by a board certified pathologist at Karolinska University Laboratory (LW). Green blocks represents homogeneous expression, while light brown colour indicates heterogeneous expression pattern across the whole tumor

Gene expression levels of *ESR1* (ER), *PGR* (PR), *ERBB2* (HER2) and *MKI67* (Ki-67) were assessed within the tumors across the spatially sampled pieces (Fig. 3a-d). *ESR1* (ER), *PGR* (PR), *ERBB2* (HER2) gene expression values tended to be homogeneous across different tumor regions, while *MKI67* mRNA levels are slightly varying between regions. Interestingly, the patient (CS-BS-00141) in which two tumor specimens with high transcriptomic grade, and two other tumor pieces had low transcriptomic grade, exhibited intra-tumor variability only in *MKI67* expression but not in *ER*, *PR* or *HER2* gene expression values (Fig. 3d). Intra-tumor variability was smaller than inter-tumor variability, and pieces from the same tumor were found to be similar on a molecular level for these biomarkers. Although we observed homogeneous mRNA levels across different regions of the same tumor, the corresponding biomarker protein (IHC based) expression (i.e. Ki-67 for *MKI67*) were more heterogeneous across spatially separated tumors (verified by board certified pathologist L.W) (Fig. 3e). PR and Ki-67 expression tended to be more spatially heterogeneous compared to ER and HER2 (Additional file 1: Figure S1). Similar findings have been reported by us and others before [34–36].

### Genomic intra-tumor heterogeneity versus molecular subtype intra-tumor heterogeneity

**Fig. 4:**
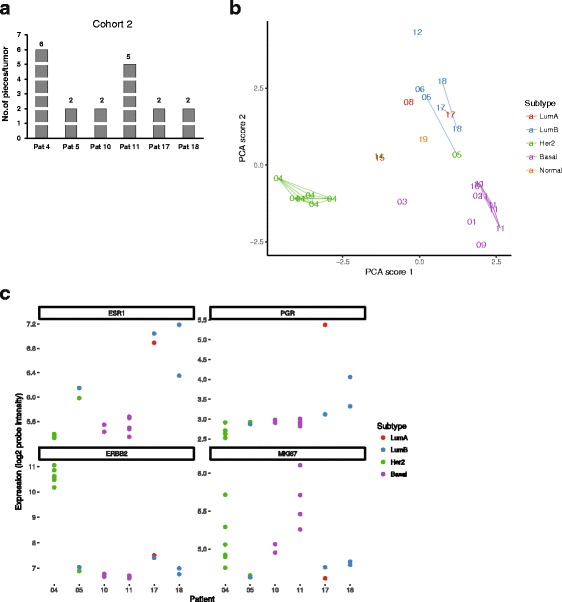
Multi-region microarray profiling from 6 breast tumors. **a** Bar graph illustrating the number of intra–tumor pieces analysed per breast tumor from 6 breast cancer patients. **b** PCA score plot of the retrospective validation cohort. Labels show patient IDs. Multiple intra-tumor pieces from the same patient are connected by lines. (Key: LumA = Luminal A (red colour), LumB = Luminal B (blue colour), HER2 = HER2-enriched (green colour), Basal = Basal-like (purple colour). **c** mRNA expression levels of *ER*, *PR*, *HER2* and *MKI67* across 19 tumor pieces from 6 multi region profiled patients

In order to validate our previous RNA sequencing based molecular profiling, we investigated 19 tumor pieces from six additional patients (2-6 pieces per tumor) and profiled them using microarray technology (Fig. 4a). Molecular subtype classification was assigned to all the tumor pieces. Analogously to the RNA-sequencing dataset, the molecular subtype remained consistent for four out of six patients when investigated across intra-tumor pieces (Fig. 4b). Two patients (patient 5 and 17) had heterogeneously classified intrinsic subtypes on spatially separated tumor samples. Patient 5 consisted of two tumor pieces, one was assigned to Luminal B and the other one to HER2-enriched subtype, while tumor pieces from patient 17 where assigned to Luminal A and Luminal B (Fig. 4b). Within each tumor, the expression levels of *ER*, *PR* and *HER2* were more or less homogenous throughout the different regions. However *MKI67* gene expression value tended to be more heterogeneous within spatially separated intratumor regions, similar to the RNA sequencing data (Fig. 4c).

**Fig. 5:**
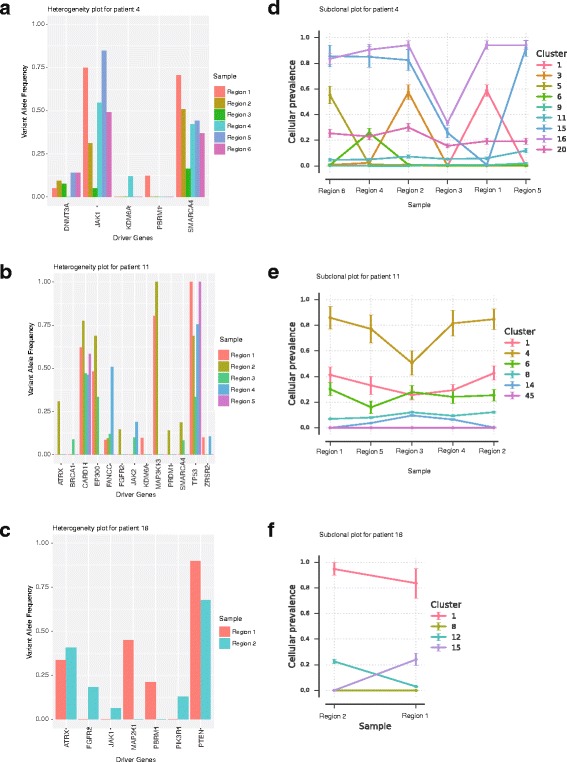
Genomic intra-tumor heterogeneity. Variant allele frequency values for putative driver genes across different regions profiled from (**a**) patient 4 (**b**) patient 11 and (**c**) patient 18. Cellular prevalence values for inferred subclones (clusters) across different regions profiled in (**d**) patient 4 (**e**) patient 11 and (**f**) patient 18

Next, we sought to investigate if intra-tumor genomic heterogeneity is common among the cases where we observed consistent molecular subtype across spatially separated tumor specimens. For this analysis, multiple regions from the six breast cancers were profiled using whole exome sequencing. We used a set of putative driver genes in breast cancer compiled by Yates et al. [37] to study intra-tumor genomic heterogeneity. In each case, we compared the driver genes, which are mutated, among different pieces from the same tumor. We observed substantial intra-tumor genomic heterogeneity in all the six patients (Fig. 5 and Additional file 2: Figure S2). For instance, intra-tumor genomic heterogeneity in patient 4, patient 11 and 18 are represented in Fig. 5 a-f. For these three patients we investigated 5, 4 and 2 tumor pieces per tumor respectively, and all the three patients retained intrinsic molecular subtype across different regions (Patient 4:HER2-enriched subtype, patient 11 basal-like and patient 18 Luminal B). In patient 4 (5 intra-tumor pieces), *PBRM* and *KDM6A* genes were mutated only in tumor piece 1 and 4 respectively but not in any of the other five tumor pieces. Further, *DNMT3A* gene was mutated in all tumor pieces except tumor piece 4 (Fig. 5a). Similarly, in patient 11, *BRCA1* was mutated only in piece 3 but not in any of the other four pieces. Few other genes such as *MAP3K13* and *JAK2* was found to be mutated only in certain tumor pieces (Fig. 5b). In patient 18, *FGFR2* was mutated online in region 2 and *MAP2K1* gene was mutated only in region 1. Few other genes such as *PTEN* and *P1K3R1* were also found to be present only in one region but not the other one (Fig. 5c). (Putative driver gene mutational differences for rest of the patients are illustrated in Additional file 2: Figure S2).

Apart from the mutational difference, subclonal analysis also identified variable contribution of tumor-related subclones in different regions in these patients. For instance, in patient 4 (Fig. 5d), out of 9 inferred subclones across six tumor pieces, subclone 1 (39 genes), subclone 3 (26 genes), subclone 5 (23 genes), and subclone 6 (29 genes) were present only in tumor piece 1, 2, 6, and 4 respectively. Subclone 15 (14 genes) was present in all tumor pieces except in tumor piece 1. Similarly in Patient 11 (Fig. 5e), six different subclones has been inferred from 5 different regions of the tumor. Subclone 14 (309 genes) was present in tumor piece 3 and 4 but not in other three tumor pieces. In patient 18 (Fig. 5f), four subclones were inferred from two different regions of the tumor, Subclone 15 (33 genes) was found to be present only in region 1, while subclone 12 (228 genes) was present in region 2. Apart from detecting subclones being present or absent, we identified variable cellular prevalence of existing subclones across different regions of a tumor for all the six patients (Additional file 2: Figure S2). Taken together, these results suggest that, substantial intra-tumor genomic heterogeneity within breast cancer is a common phenomenon, however, this intra-tumor genetic heterogeneity does not affect the molecular subtype classifications to a great extent.

## Discussion

Spatial tumor heterogeneity impacts traditional immunohistochemical analysis. Variations in ER, PR and HER2 expression in spatially separated tumor samples has been reported before and is sometimes associated with heterogeneity in morphology [35, 38, 39]. Proliferation markers such as Ki-67 are also subjected to substantial intra-tumor heterogeneity [36] with higher expression in certain hot-spots and in the tumor invasive margins [34]. Determining the tumor grade and molecular subtype by IHC surrogate classification are highly sensitive to the cut-off of the Ki-67 score and the region of the tumor investigated [40, 41]. Further, inter-individual variability between pathologists also accounts for misclassification of tumors [42, 43]. Therefore, next generation technologies such as automatic image processing technology, gene expression based molecular profiling and genetic testing are considered as the future of cancer diagnostics. In order to translate such technologies to the clinic, they should be sufficiently robust and consistent in providing therapy predictive and prognostic information without being affected by typical levels of intra-tumor heterogeneity.

In this study we focused on assessing if the sampling procedure, i.e. which part of the tumor to profile by RNA-sequencing, would have an impact on transcription-based molecular breast cancer diagnostics. RNA-sequencing based transcriptomic profiling of multiple pieces from the same tumors (*n* = 12, Cohort 1) revealed minor intra-tumor differences. Only one patient (CS-BC-00059) exhibited heterogeneous molecular subtype and one patient (CS-BS-00141) demonstrated heterogeneous transcriptomic grade scores in a cohort of 12 breast tumors. In both the patients, *ER*, *PR* and *HER2* expression remained homogenous across tumor pieces, while, *MKI67* expression varied in spatially separated tumor pieces. We observed similar findings in all the 12 patients, where *MKI67* tend to be more intra-tumoral heterogeneous compared to *ER*, *PR* and *HER2*. This suggests that proliferation markers such as *MKI67* are crucial factor that influence molecular subtype and transcriptomic grade heterogeneity. On the other hand, protein expression of ER, PR, HER2 and Ki-67 exhibited more spatial heterogeneity than mRNA levels. In our study, we observed that, PR and Ki-67 protein levels tend to be more heterogeneous than ER and HER2 protein expression. Further, we applied microarray-based gene expression profiling in an independent cohort (Cohort 2), which consisted of multiple regions from six primary breast tumors (19 pieces in total) to validate the molecular subtype homogeneity between intra-tumor pieces. We assigned molecular subtypes to each region and found that molecular subtypes were consistent between tumor pieces in four out of six patients. In two patients (patient 5 and 17) however, we observed heterogeneous molecular subtypes between two pieces of the same tumor. Similar to previous cohort, *MKI67* expression tends to me more heterogeneous across intra-tumor pieces than *ER*, *PR* and *HER2* expression.

It has been well established that substantial intra-tumor genomic differences are common in breast cancer [21, 22, 37]. In our cohort, we observed intra-tumor genomic heterogeneity in all the six patients. However, we observed homogeneous molecular subtype between intra-tumor pieces. Our subclonal analysis also revealed that certain subclones are only present in certain parts of the tumor. However, their cellular prevalence is much lower compared to the dominant clones of the tumor. It is possible for small subclones within the tumor to have radically different molecular make up when compared to the rest of tumor bulk. However, current clinical practices are mostly administrated based on the molecular characteristics of the entire tumor, while there is a risk of a future recurrence due to expansion of the minor (undetected) subclone during cancer progression. For instance, we and others have previously reported that the expression of prognostic and therapy-predictive biomarkers were altered in metastasis compared to their respective primary tumors, this might be due to the undetected subclone in primary tumors which could have expanded in metastasis during tumor evolution [44, 45].

There are multiple limitations in our study, primarily the sample size, uneven molecular subtype distribution among the samples and lack of relative spatial information (physical distance measurements) between regions analysed within each tumor. It might well be that for some smaller proportion of patients; intra-tumor heterogeneity may be of importance, while substantially larger studies would be required to establish if this is the case, particularly if the proportion of tumor with large intra-tumor heterogeneity is small. In this study the number of regions analysed within each tumor depends on the initial size of the whole tumor, and the resolution of the intra-tumor heterogeneity is characterised on a macroscopic scale rather than a microscopic scale. The application of e.g. single-cell gene expression profiling or similar technologies could potentially be applied in future studies to characterise intra-tumor heterogeneity at a higher resolution. Further, we could not determine the protein expression, using e.g. IHC, of therapeutic predictive markers (ER, PR, and HER2) from the same regions of tumor pieces that were used for RNA-sequencing. However, previous studies have reported substantial protein expression heterogeneity within the breast tumors [35].

## Conclusions

In summary, this study demonstrates that the average expression profile collected from any part of the breast tumor in most cases is representative for the entire tumor, at least with respect to transcriptomic grade and molecular subtype. Further, the variability introduced by random sampling of material from the tumor is not expected to have a major impact for most patients, even though these intra-tumor pieces demonstrates substantial spatial genomic heterogeneity.

## Additional files

Additional file 1: Figure S1. Representative immunohistochemical staining (IHC) images of heterogeneous expression patterns of **(a)** ER **(b)** PR **(c)** HER2 and **(d)** Ki-67 in two patients. Scale bar = 200 μm. Regions with higher protein expression are marked with red arrows and regions with lower protein staining are marked in green arrows. (PDF 568 kb)

Additional file 2: Figure S2. Variant allele frequency values for putative driver genes across different regions profiled from **(a)** patient 15 **(b)** patient 10 and **(c)** patient 17. Cellular prevalence values for inferred subclones (clusters) across different regions profiled in **(d)** patient 5 **(e)** patient 10 and **(f)** patient 17. (PDF 1153 kb)

## Abbreviations

ER: Estrogen receptor
FFPE: Formalin-fixed paraffin-embedded
HER2: Human epidermal growth factor receptor 2
IHC: Immunohistochemistry
NHG: Nottingham histological grade
PCA: Principal Component Analysis
PR: Progesterone receptor
TG: Transcriptomic grade
VAF: Variant allele frequency

## References

[1] Jatoi I, Miller AB (2003). Why is breast-cancer mortality declining?. The Lancet Oncology.

[2] Bastien RRL, Rodriguez-Lescure A, Ebbert MTW, Prat A, Munarriz B, Rowe L, Miller P, Ruiz-Borrego M, Anderson D, Lyons B (2012). PAM50 breast cancer subtyping by RT-qPCR and concordance with standard clinical molecular markers. BMC Med Genet.

[3] Parker JS, Mullins M, Cheang MC, Leung S, Voduc D, Vickery T, Davies S, Fauron C, He X, Hu Z (2009). Supervised risk predictor of breast cancer based on intrinsic subtypes. J Clin Oncol.

[4] Sorlie T, Perou CM, Tibshirani R, Aas T, Geisler S, Johnsen H, Hastie T, Eisen MB, van de Rijn M, Jeffrey SS (2001). Gene expression patterns of breast carcinomas distinguish tumor subclasses with clinical implications. Proc Natl Acad Sci U S A.

[5] Sorlie T, Tibshirani R, Parker J, Hastie T, Marron JS, Nobel A, Deng S, Johnsen H, Pesich R, Geisler S (2003). Repeated observation of breast tumor subtypes in independent gene expression data sets. Proc Natl Acad Sci U S A.

[6] Lyman GH, Cosler LE, Kuderer NM, Hornberger J (2007). Impact of a 21-gene RT-PCR assay on treatment decisions in early-stage breast cancer: an economic analysis based on prognostic and predictive validation studies. Cancer.

[7] Wallden B, Storhoff J, Nielsen T, Dowidar N, Schaper C, Ferree S, Liu S, Leung S, Geiss G, Snider J (2015). Development and verification of the PAM50-based Prosigna breast cancer gene signature assay. BMC Med Genet.

[8] Wittner BS, Sgroi DC, Ryan PD, Bruinsma TJ, Glas AM, Male A, Dahiya S, Habin K, Bernards R, Haber DA (2008). Analysis of the MammaPrint breast cancer assay in a predominantly postmenopausal cohort. Clinical cancer research : an official journal of the American Association for Cancer Research.

[9] Cardoso F, van’t Veer LJ, Bogaerts J, Slaets L, Viale G, Delaloge S, Pierga JY, Brain E, Causeret S, DeLorenzi M (2016). 70-gene signature as an aid to treatment decisions in early-stage breast cancer. N Engl J Med.

[10] Rantalainen M, Klevebring D, Lindberg J, Ivansson E, Rosin G, Kis L, Celebioglu F, Fredriksson I, Czene K, Frisell J (2016). Sequencing-based breast cancer diagnostics as an alternative to routine biomarkers. Sci Rep.

[11] Wang M, Klevebring D, Lindberg J, Czene K, Gronberg H, Rantalainen M (2016). Determining breast cancer histological grade from RNA-sequencing data. Breast cancer research : BCR.

[12] Sotiriou C, Wirapati P, Loi S, Harris A, Fox S, Smeds J, Nordgren H, Farmer P, Praz V, Haibe-Kains B (2006). Gene expression profiling in breast cancer: understanding the molecular basis of histologic grade to improve prognosis. J Natl Cancer Inst.

[13] Ivshina AV, George J, Senko O, Mow B, Putti TC, Smeds J, Lindahl T, Pawitan Y, Hall P, Nordgren H (2006). Genetic reclassification of histologic grade delineates new clinical subtypes of breast cancer. Cancer Res.

[14] Sotiriou C, Piccart MJ (2007). Taking gene-expression profiling to the clinic: when will molecular signatures become relevant to patient care?. Nat Rev Cancer.

[15] Wennmalm K, Bergh JA (2011). Simple method for assigning genomic grade to individual breast tumours. BMC Cancer.

[16] Polyak K (2011). Heterogeneity in breast cancer. J Clin Invest.

[17] Marusyk A, Almendro V, Polyak K (2012). Intra-tumour heterogeneity: a looking glass for cancer?. Nat Rev Cancer.

[18] Martelotto LG, Ng CK, Piscuoglio S, Weigelt B, Reis-Filho JS (2014). Breast cancer intra-tumor heterogeneity. Breast Cancer Res.

[19] Elston CW, Ellis IO (1991). Pathological prognostic factors in breast cancer. I. The value of histological grade in breast cancer: experience from a large study with long-term follow-up. Histopathology.

[20] Denisov EV, Litviakov NV, Zavyalova MV, Perelmuter VM, Vtorushin SV, Tsyganov MM, Gerashchenko TS, Garbukov EY, Slonimskaya EM, Cherdyntseva NV (2014). Intratumoral morphological heterogeneity of breast cancer: neoadjuvant chemotherapy efficiency and multidrug resistance gene expression. Sci Rep.

[21] Navin N, Krasnitz A, Rodgers L, Cook K, Meth J, Kendall J, Riggs M, Eberling Y, Troge J, Grubor V (2010). Inferring tumor progression from genomic heterogeneity. Genome Res.

[22] Ding L, Ellis MJ, Li S, Larson DE, Chen K, Wallis JW, Harris CC, McLellan MD, Fulton RS, Fulton LL (2010). Genome remodelling in a basal-like breast cancer metastasis and xenograft. Nature.

[23] Navin N, Kendall J, Troge J, Andrews P, Rodgers L, McIndoo J, Cook K, Stepansky A, Levy D, Esposito D (2011). Tumour evolution inferred by single-cell sequencing. Nature.

[24] Shah SP, Roth A, Goya R, Oloumi A, Ha G, Zhao Y, Turashvili G, Ding J, Tse K, Haffari G (2012). The clonal and mutational evolution spectrum of primary triple-negative breast cancers. Nature.

[25] Jiang H, Lei R, Ding SW, Zhu S (2014). Skewer: a fast and accurate adapter trimmer for next-generation sequencing paired-end reads. BMC bioinformatics.

[26] Dobin A, Davis CA, Schlesinger F, Drenkow J, Zaleski C, Jha S, Batut P, Chaisson M, Gingeras TRSTAR (2013). ultrafast universal RNA-seq aligner. Bioinformatics.

[27] Anders S, Pyl PT, Huber W (2015). HTSeq-a python framework to work with high-throughput sequencing data. Bioinformatics.

[28] Robinson MD, Oshlack A (2010). A scaling normalization method for differential expression analysis of RNA-seq data. Genome Biol.

[29] Robinson MD, McCarthy DJ, Smyth GK (2010). edgeR: a bioconductor package for differential expression analysis of digital gene expression data. Bioinformatics.

[30] R Core Team RFfSC (2016). R: a language and environment for statistical computing.

[31] Zhao X, Rodland EA, Tibshirani R, Plevritis S (2015). Molecular subtyping for clinically defined breast cancer subgroups. Breast Cancer Res.

[32] Pawitan Y, Bjohle J, Amler L, Borg AL, Egyhazi S, Hall P, Han X, Holmberg L, Huang F, Klaar S (2005). Gene expression profiling spares early breast cancer patients from adjuvant therapy: derived and validated in two population-based cohorts. Breast Cancer Res.

[33] Ovaska K, Laakso M, Haapa-Paananen S, Louhimo R, Chen P, Aittomaki V, Valo E, Nunez-Fontarnau J, Rantanen V, Karinen S (2010). Large-scale data integration framework provides a comprehensive view on glioblastoma multiforme. Genome medicine.

[34] Stalhammar G, Fuentes Martinez N, Lippert M, Tobin NP, Molholm I, Kis L, Rosin G, Rantalainen M, Pedersen L, Bergh J (2016). Digital image analysis outperforms manual biomarker assessment in breast cancer. Mod Pathol.

[35] Allott EH, Geradts J, Sun X, Cohen SM, Zirpoli GR, Khoury T, Bshara W, Chen M, Sherman ME, Palmer JR (2016). Intratumoral heterogeneity as a source of discordance in breast cancer biomarker classification. Breast cancer research : BCR.

[36] Besusparis J, Plancoulaine B, Rasmusson A, Augulis R, Green AR, Ellis IO, Laurinaviciene A, Herlin P, Laurinavicius A (2016). Impact of tissue sampling on accuracy of Ki67 immunohistochemistry evaluation in breast cancer. Diagn Pathol.

[37] Yates LR, Gerstung M, Knappskog S, Desmedt C, Gundem G, Van Loo P, Aas T, Alexandrov LB, Larsimont D, Davies H (2015). Subclonal diversification of primary breast cancer revealed by multiregion sequencing. Nat Med.

[38] Layfield LJ, Saria E, Mooney EE, Liu K, Dodge RR (1998). Tissue heterogeneity of immunohistochemically detected estrogen receptor. Implications for image analysis quantification. Am J Clin Pathol.

[39] Buckley NE, Forde C, McArt DG, Boyle DP, Mullan PB, James JA, Maxwell P, McQuaid S, Salto-Tellez M (2016). Quantification of HER2 heterogeneity in breast cancer-implications for identification of sub-dominant clones for personalised treatment. Sci Rep.

[40] Dowsett M, Nielsen TO, A'Hern R, Bartlett J, Coombes RC, Cuzick J, Ellis M, Henry NL, Hugh JC, Lively T (2011). Assessment of Ki67 in breast cancer: recommendations from the international Ki67 in breast cancer working group. J Natl Cancer Inst.

[41] Focke CM, van Diest PJ, Decker T (2016). St Gallen 2015 subtyping of luminal breast cancers: impact of different Ki67-based proliferation assessment methods. Breast Cancer Res Treat.

[42] Elmore JG, Longton GM, Carney PA, Geller BM, Onega T, Tosteson AN, Nelson HD, Pepe MS, Allison KH, Schnitt SJ (2015). Diagnostic concordance among pathologists interpreting breast biopsy specimens. JAMA.

[43] Bueno-de-Mesquita JM, Nuyten DS, Wesseling J, van Tinteren H, Linn SC, van de Vijver MJ (2010). The impact of inter-observer variation in pathological assessment of node-negative breast cancer on clinical risk assessment and patient selection for adjuvant systemic treatment. Annals of oncology : official journal of the European Society for Medical Oncology / ESMO.

[44] Karlsson E, Sandelin K, Appelgren J, Zhou W, Jirstrom K, Bergh J, Warnberg F (2014). Clonal alteration of breast cancer receptors between primary ductal carcinoma in situ (DCIS) and corresponding local events. Eur J Cancer.

[45] Lindstrom LS, Karlsson E, Wilking UM, Johansson U, Hartman J, Lidbrink EK, Hatschek T, Skoog L, Bergh J (2012). Clinically used breast cancer markers such as estrogen receptor, progesterone receptor, and human epidermal growth factor receptor 2 are unstable throughout tumor progression. Journal of clinical oncology : official journal of the American Society of Clinical Oncology.

