# Supplementary figures and images for: Intra-tumor heterogeneity in breast cancer has limited impact on transcriptomic-based molecular profiling
Source: BMC Cancer. 2017 Nov 29;17:802. doi: 10.1186/s12885-017-3815-2 (PMC5708109; doi:10.1186/s12885-017-3815-2)

Additional file 1: Figure S1:

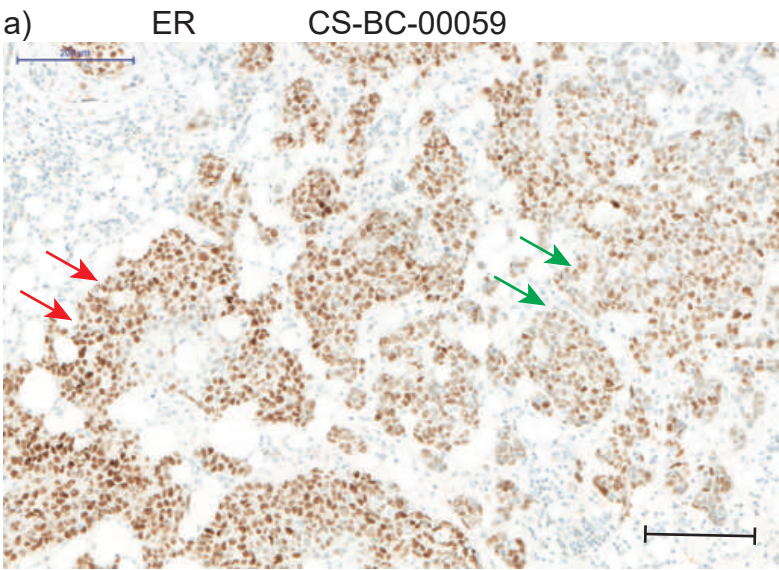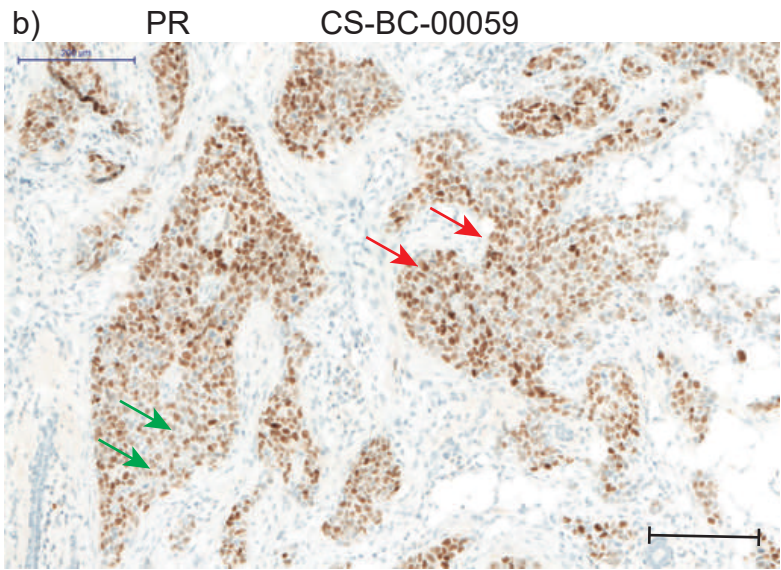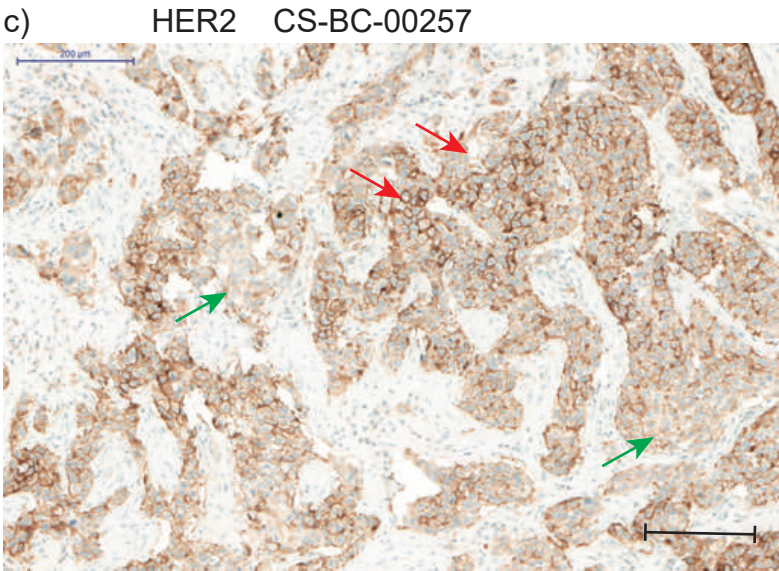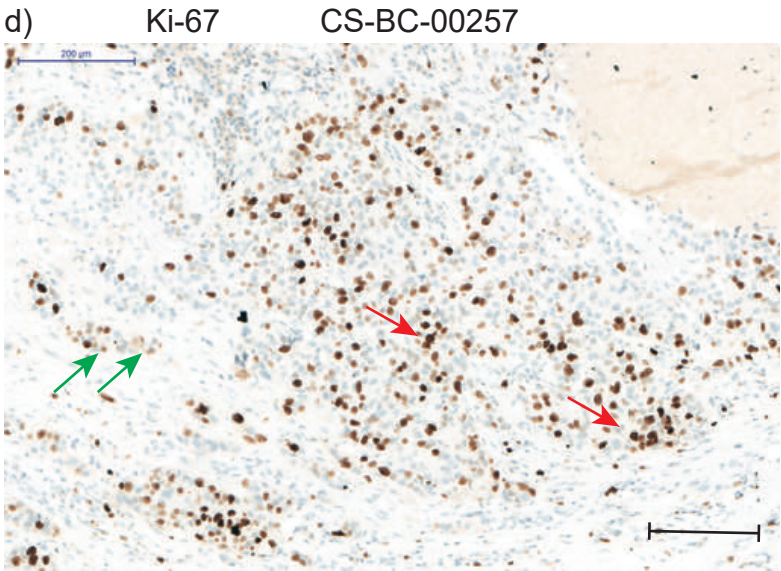

Supplement: Supplementary file 1 — Representative immunohistochemical staining (IHC) images of heterogeneous expression patterns of (a) ER (b) PR (c) HER2 and (d) Ki-67 in two patients. Scale bar = 200 μm. Regions with higher protein expression are marked with red arrows and regions with lower protein staining are marked in green arrows. (PDF 568 kb) [file 12885_2017_3815_MOESM1_ESM.pdf]

Additional file 2: Figure S2:

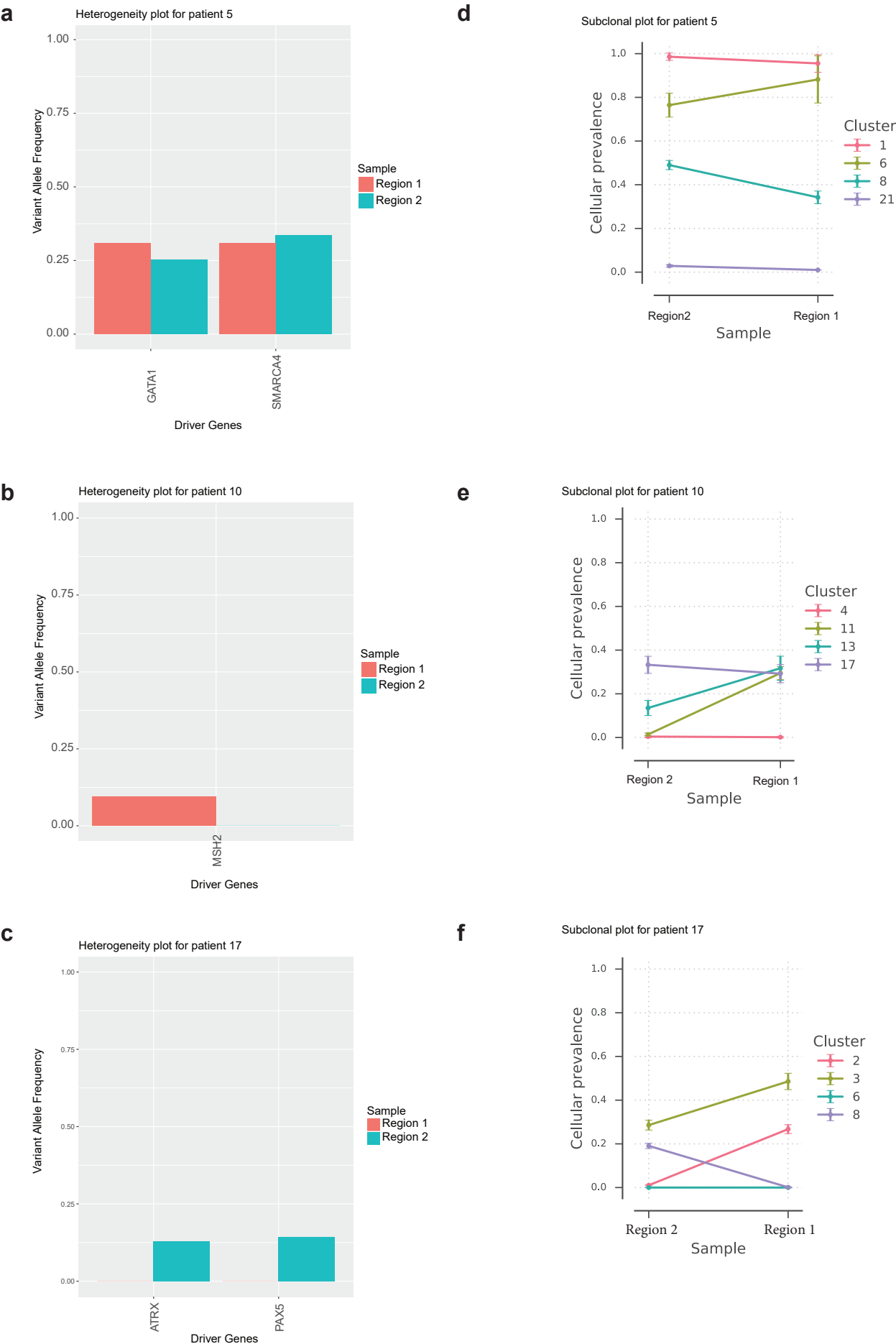

Supplement: Supplementary file 2 — Variant allele frequency values for putative driver genes across different regions profiled from (a) patient 15 (b) patient 10 and (c) patient 17. Cellular prevalence values for inferred subclones (clusters) across different regions profiled in (d) patient 5 (e) patient 10 and (f) patient 17. (PDF 1153 kb) [file 12885_2017_3815_MOESM2_ESM.pdf]
